# Metaphorical discourse in Beijing Winter Olympic news: a Trinocular Perspective analysis of language, cognition, and social functions

**DOI:** 10.3389/fpsyg.2024.1477890

**Published:** 2024-12-17

**Authors:** Wei Peng, Qingping Li

**Affiliations:** School of Foreign Languages, Central South University, Changsha, China

**Keywords:** Olympic news, metaphor, Trinocular Perspective, cognition, social function

## Abstract

This study utilizes the “Trinocular Perspective” metaphor analysis framework to delve into the language, cognition, and social functions of metaphors in news coverage of the Beijing Winter Olympics and their interactions, thereby revealing the role of metaphors in shaping and articulating the narratives within Olympic news communication. The research indicated that, at the linguistic level, metaphors enhanced the expressiveness and appeal of news discourse through diverse forms and dense distribution. At the cognitive level, metaphors fostered a deeper public understanding of abstract concepts such as the Olympic spirit, the values of a community with a shared future for mankind, China’s national image, and the concept of sustainable development. On the social function level, metaphors will play a pivotal role in building social identity, disseminating values, highlighting social issues, and promoting social change. The language, cognition, and social functions of metaphors were closely interwoven, with linguistic expressions enriching cognitive frameworks, cognitive frameworks guiding the internalization and dissemination of social values, and social functions guiding public cognition through the strategic use of metaphorical language. This study reveals the critical function of metaphors in elevating the narrative richness and dissemination efficacy within the journalism of large-scale international sports competitions.

## Introduction

The strategic deployment of metaphors in sports news communication is a prevalent and powerful practice, particularly within the coverage of major international events like the Olympics (Ndegwa, 2016; Ross and Rivers, 2019; Yu, 2011). Metaphors are not just linguistic tools but are instrumental in shaping public perceptions and conveying values (Cotter et al., 2021; Kazemian and Hatamzadeh, 2022; Yu, 2021). The Beijing Winter Olympics, as the first “Dual Olympics City” in the world, presented a unique scenario where official media extensively employed metaphors to embellish language, sculpt public views on the Winter Olympics and China, and disseminate cultural values. This underscores the critical function of metaphors in mediating our understanding and appreciation of such grand sporting events.

Metaphor, as a complex linguistic phenomenon, transcends mere rhetorical devices and plays a core role in human cognition and interaction processes (Lakoff and Johnson, 1980). With the deepening of research, metaphor has been recognized as having linguistic, cognitive, and social properties, making it a multidimensional research object (Gibbs, 1994). To elucidate the roles played by the three aspects of metaphor, scholars have successively proposed theoretical frameworks such as the Three-Dimensional Model and the “Trinocular Perspective” (Steen, 2008; He, 2016). The “Trinocular Perspective” framework has guided metaphor research from theoretical exploration to practical application, providing a highly significant and operational research paradigm for the study of metaphors. However, the “Trinocular Perspective” framework still needs more empirical verification through large-scale news corpus research methods. Additionally, more research is needed to explore the interplay between the linguistic, cognitive, and socio-communicative dimensions of metaphors, as they are not isolated but interact and influence each other in complex ways.

Metaphors on the linguistic level convey not only messages but also reflect cognitive structures that are disseminated through social interaction, which in turn affects language use. Therefore, clarifying the interplay among these dimensions and elucidating their operational mechanisms in practical discourse is crucial for understanding and applying metaphors in real-world contexts. This study employs the “Trinocular Perspective” analytical framework to study the metaphors in the news discourse of the Beijing Winter Olympics, revealing how metaphorical language constructs narratives, shapes conceptual understanding at the cognitive level, and promotes social and cultural exchange at the socio-functional level, as well as the mechanisms of their interaction. Such analysis not only reveals media strategies but also provides a novel perspective on the role of metaphor in contemporary news dissemination.

## Literature review

### Three attributes of metaphor

Metaphor is a rich linguistic phenomenon that encompasses cognitive, linguistic, and socio-communicative dimensions, making it a multifaceted tool for thought and expression. From a cognitive perspective, metaphors play a central role in our understanding of abstract concepts, typically shaping cognitive processes by mapping familiar, concrete concepts (source domains) onto unfamiliar, abstract concepts (target domains). Aristotle regarded metaphor as indicative of genius, as it necessitates the recognition of similarities between distinct entities (Barnes, 1984). Subsequently, metaphor has been recognized as a cognitive instrument, giving rise to the concept of “metaphorical thought” (Black, 1962). Richards (1965) was the first to systematically elaborate on the cognitive nature of metaphor, considering it an integral part of our thought patterns. Lakoff and Johnson (1980) seminal work, “Metaphors We Live By,” ushered metaphor research into the realm of cognitive science.

The linguistic attributes of metaphor are also indispensable, influencing how we interpret and understand language through structure, vocabulary, and rhetoric. While early research focused on these manifestations, the rise of conceptual metaphor theory shifted the focus towards metaphor’s cognitive underpinnings (Lakoff, 1993; Lakoff and Johnson, 1999). Afterwards, researchers have reassessed the linguistic properties of metaphor, highlighting the importance of metaphor research based on authentic discourse (Aloairdhi and Kahlaoui, 2020; Goatly, 1997; Knowles and Moon, 2006). Cameron (2003) noted that metaphor research must concurrently address both linguistic and cognitive dimensions, advocating for an integrated approach to the theoretical research and analysis of metaphor.

Lastly, the socio-communicative function of metaphor is another key attribute. While contemporary metaphor theory has concentrated on the nature and functions of language and thought, the socio-communicative aspect of metaphor has not received ample attention. Studies indicated that metaphor does not appear in isolation within discourse but interacts with sociocultural factors, facilitating communicative goals (Charteris-Black, 2004; Thibodeau et al., 2019). Research on the social attribute of metaphor explained how individuals use it as a tool to “construct social reality,” express personal attitudes, emotions, and values, and achieve social interaction (Gutiérrez Escalante, 2019; Zeng et al., 2020). The communicative aspect of metaphor is often explained through conceptual and linguistic characteristics (Kövecses, 2002). Thus, the cognitive, linguistic, and socio-communicative attributes of metaphor typically coexist and interact within a given context. Nevertheless, the manner in which these three attributes synergistically function and influence one another remains to be further explored.

### The three-dimensional model of metaphor

To more comprehensively elucidate the roles of metaphor in language, cognition, and social communication, Gerard Steen have proposed a Three-Dimensional Model of metaphor (Steen, 2008; Steen, 2011). This model extends beyond the cognitive and linguistic functions of metaphor to include the dimension of communication. Gerard Steen’s three-dimensional metaphor model encompasses three core dimensions (Steen, 2008; Steen, 2011) the language dimension, which focuses on the forms of metaphor in language expression, including metaphors and analogies as rhetorical devices and their application within sentence structures; the thought dimension, which explores how metaphor operates across different domains in cognition, connecting one conceptual field to another to facilitate understanding and thought; and the communication dimension, which analyzes the strategic use of metaphor in actual communication, particularly how speakers employ metaphor to influence listeners’ perspectives or emotions. Steen posited that these three dimensions were both distinct and interrelated. For instance, the metaphor “he is as brave as a lion” linguistically compares a person to a lion, cognitively maps the qualities of strength and courage, and communicatively may emphasize the individual’s brave qualities. Steen’s model transcends the two-dimensional view of conceptual metaphor theory (Lakoff, 1993; Lakoff and Johnson, 1980), highlighting the significance of the communicative dimension and enabling a more nuanced analysis of the multiple roles metaphor plays in various contexts and its impact on cognitive and communicative processes.

The Three-Dimensional Model offers a novel perspective for exploring the role of metaphor in social interaction, prompting scholars to delve into its functions across linguistic, cognitive, and communicative dimensions. In everyday communication, metaphors not only exist as linguistic phenomena but also reflect speakers’ cognitive patterns and communicative intentions (Rewiś-Łętkowska, 2019). Metaphor’s complex impact on cognitive, discursive, and practical levels is evident when addressing sensitive topics such as names of diseases. Semino et al. (2016) uncovered the intricacies of metaphor in shaping individual and collective attitudes towards cancer, emphasizing its critical role in both cognitive processes and actual communication, providing valuable insights for health communication practices. In discussions of social issues, the application of metaphor is equally significant. Zeng and Ahrens (2023) analyzed the war metaphors in Hong Kong public discourse, finding that social issues are primarily understood through a combat framework, while economic issues are more often discussed through a strategic framework. In the realm of political communication, the Three-Dimensional Model has been expanded to analyze the deliberate use of metaphor in non-institutional political interviews. Heyvaert et al. (2019) found that metaphors in interviews manifest directly or indirectly express ideas, map political concepts cognitively, and shape the audience’s political understanding communicatively. Additionally, the Three-Dimensional Model can be integrated with argumentation theory to uncover the multidimensional manifestations of metaphor within argumentative discourse (Van Poppel, 2021).

In summary, the application of the Three-Dimensional Model demonstrated that the roles of metaphor in language, cognition, and socio-communicative contexts were complex and multifaceted. While the Three-Dimensional Model of metaphor provides a macro framework for understanding the function of metaphor in three dimensions, there is a need to develop a more detailed methodology for analyzing the specific mechanisms of metaphor in actual context. In other words, it does not offer a tangible research paradigm for applying this model in empirical studies, particularly when dealing with large corpora and complex sociocultural backgrounds.

### The “Trinocular Perspective” analysis of metaphor

To enhance the application of the Three-Dimensional Model, scholars have proposed the “Trinocular Perspective” analysis framework for metaphor. The “Trinocular Perspective” is a core concept in systemic functional linguistics. Halliday (1996) suggested that grammatical categories can be described from three angles: the semantic perspective from “above” examines the expression of meaning; the lexical perspective from “roundabout” analyzes relationships between grammatical categories; and the morphological perspective from “below” deconstructs the manifestation of language. Matthiessen et al. (2010) noted that this perspective was not only for describing grammatical categories but also served as a general method for multi-dimensional language research. This perspective aligns with the cognitive, linguistic, and social attributes of metaphor, allowing for an organic integration of conceptual metaphor with contextual factors. Based on this, He (2016) restructured and expanded the three levels and trinocular structure to propose the “Trinocular Perspective” analysis framework for metaphor, suggesting that metaphor analysis should be conducted from three angles: the “from below” angle focuses on linguistic metaphors, exploring their linguistic attributes such as metaphorical constructions, density, and clusters; the “from roundabout” angle takes conceptual metaphors as the object, discussing cognitive attributes through metaphor networks and scenarios or stories; and the “from above” angle concentrates on the user identity and functions of metaphor, elucidating its social attributes. This Trinocular approach extends the Three-Dimensional Model, offering specific tools and methods for practical analysis of each dimension, guiding metaphor research from theoretical to practical applications, and enabling researchers to more precisely reveal how metaphor functions on different levels. Consequently, the “Trinocular Perspective” framework not only compensates for the shortcomings of the Three-Dimensional Model but also advances the methodology of metaphor analysis, facilitating in-depth exploration of the role and impact of metaphor across cognitive science, linguistics, and social sciences.

Some scholars have utilized the “Trinocular Perspective” framework for metaphor analysis in corpus studies. Huo and Liu (2018) combined this framework with framing theory to analyze the conceptual metaphors in the political speeches of U.S. presidential candidates from both parties on the issue of climate change. They examined the superficial structures from the “from below” perspective, delved into deeper structures and metaphorical thinking from the “from roundabout” perspective, and analyzed mapping pathways and metaphor functions from the “from above” perspective. Through comparative analysis, they revealed the differences in the stances of Democrats and Republicans on climate change and how these positions were reflected in political speeches. Thus, the “Trinocular Perspective” analysis of metaphor is applicable in media discourse analysis and political oratory.

However, the interactions among language, cognition, and social functions within the “Trinocular Perspective” framework have not been adequately addressed, particularly the systematic analysis of their interplay in real-world social contexts. Although Steen’s Three-Dimensional Model notes that the three dimensions influence each other, it does not elaborate on this interaction. Moreover, despite the theoretical potential of the “Trinocular Perspective” framework, its combination with large-scale news corpus research methods has not been sufficiently empirically tested. Such an integrated research approach could provide a systematic, comprehensive, and precise pathway for analyzing news discourse of major international sporting events like the Beijing Winter Olympics, offering a practical case for exploring the interaction between language, cognition and social functions.

### Media discourse in the Beijing Winter Olympics

The historical backdrop of the Beijing Winter Olympics is deeply rooted in China’s emergence as a global sports powerhouse and its desire to showcase its comprehensive national strength and cultural charm on the international stage. The Beijing Winter Olympics, as a global sports event, has inspired in-depth research and discussions on China’s national image, social reality, and sustainable development. The successful hosting of the Games was regarded as an important opportunity for China to showcase its national image and enhance its international status. Cui (2023) utilized the Discourse-Historical Approach (DHA) and corpus techniques to analyze the strategies adopted by People’s Daily Online in constructing China’s national image. The study indicated that through the use of discursive strategies, China was portrayed as a peaceful, responsible, environmentally conscious, open, fair, and cooperative nation. Shi and Zhang (2022) explored the impact of mediatization on the Beijing 2022 Winter Olympics and how the public received this mediatized process. By analyzing WeChat posts and conducting semi-structured interviews, the study found that technological advancement and the “Green Olympics” were the two major themes in media coverage, closely related to China’s national agenda. Zhang et al. (2023) compared the reporting strategies of People’s Daily and The New York Times on the Beijing Winter Olympics, revealing differences in the mainstream media of China and the United States. They found that Chinese media tend to present the positive aspects of the nation, emphasizing technological innovation and international cooperation, while American media focus more on political positions and ideological differences. The study also pointed out that understanding the different strategies of Chinese and American media in reporting the Winter Olympics helps to promote mutual communication between the two countries, eliminate misunderstandings, and jointly create a favorable international communication environment. Boykoff (2024) focused on the reporting frames of American media for the Beijing Winter Olympics, noting that American media demonstrated clear political bias and framing effects in their coverage, not only focusing on the sports themselves but also on the complexity of political, social, and cultural aspects behind the events. This mode of reporting resulted in the audience’s understanding of the event being confined to the narrative frames set by the media.

In summary, research related to the Beijing Winter Olympics spans various dimensions, including media discourse construction, international communication differences, dissemination of technology and green environmental protection concepts, and media reporting frame analysis, showcasing the multiple significances of the Beijing Winter Olympics under different cultural, political, and media backgrounds. Building on this foundation, this paper will employ a “Trinocular Perspective” analytical framework to further explore the linguistic expression, cognitive framework, and social function of metaphors in news reporting, and to analyze how these aspects interact to influence public cognition and attitude. Through this research, we expect to provide a deeper understanding of the practical application of metaphors in real-world contexts and offer new research pathways for the field of sports news communication.

## Methodology

**Figure 1:**
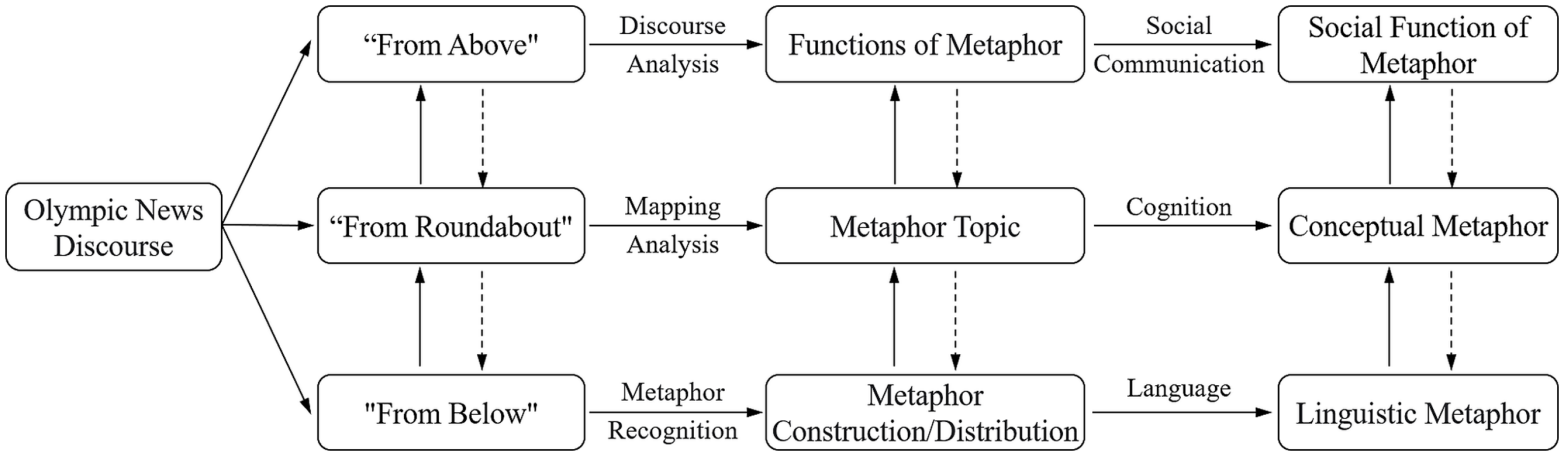
Analytical framework of this study.

Leveraging the robust comprehensive analytical capabilities of the “Trinocular Perspective,” this paper applied the Trinocular framework to thoroughly examine the use of metaphor in Beijing Winter Olympics news discourse and reveal how metaphors interact across linguistic, cognitive, and social dimensions. The analytical framework of this study is illustrated in Figure 1. From the “from below” linguistic perspective, the study focuses on the construction and distribution of metaphors and their specific application within news reports. This viewpoint will uncover how metaphors are effectively encoded and conveyed within news texts. From the “from roundabout” cognitive perspective, the focus is on the target domains and metaphor topic, exploring how metaphors facilitate the construction and understanding of abstract concepts such as the Olympic spirit and national image through cross-domain mapping. This analysis will delve into the internal structure of metaphors, revealing how they promote cognitive processes and conceptual understanding. Finally, from the “from above” social functional perspective, the study examines the role of metaphors within discourse, investigating how they function in social communication, including their roles in building social identity, disseminating values, and highlighting social issues. This analysis provides insights into how metaphors serve as powerful tools for communication and influencing public opinion in real social interactions.

### Retrieval and selection of news reports

This study focuses on Winter Olympics coverage from major Chinese media outlets: “People’s Daily,” “China Daily,” and “Xinhua Net,” all of which are influential party media outlets in China (Yang et al., 2023). To present a comprehensive view of the Beijing Winter Olympics, news reports from the entire period of the Games, February 4th to February 20th, 2022, were collected. Using “Beijing Winter Olympics” as the keyword, searches were conducted in the official databases of these media outlets, with a focus on relevance in both form and content. The criteria for selection were: 1. A minimum of 500 words in the report’s body; 2. High relevance to the Winter Olympics, including events, athletes, ceremonies, venues, and infrastructure, while excluding less relevant topics such as insurance, finance, and healthcare. A total of 181 news reports, totaling 230,554 words, were selected, with 63 from “People’s Daily,” 71 from “China Daily,” and 47 from “Xinhua Net,” to form the media discourse corpus for the Beijing Winter Olympics.

### Identification and analysis of metaphors

The integration of corpus analysis with metaphor theory is a prevalent trend in metaphor research (Han, 2014). The Metaphor Identification Procedure (MIP) is a systematic approach for detecting metaphorical expressions in discourse, determining whether a specific lexical unit in spoken or written language can be identified as a metaphor within a given context (Pragglejaz Group, 2007). This study utilized the MIP in conjunction with AntConc 3.5.9 (2020 version) for metaphor detection (Luo and Zhai, 2017). The process included: 1. Read the entire news reports in the corpus to understand the overall message; 2. Extracted thematic words from the news reports and generating a frequency list using AntConc; 3. Comprehensively searched for the contextual meanings of these thematic words to ascertain their situational meanings, and comparing these with their basic meanings from the “Modern Chinese Dictionary” to identify and mark metaphorical keywords; 4. Analyzed the frequency of these metaphorical keywords and categorizing them based on their source domains.

For the induction of metaphor topics, the identified keywords were sorted into 11 sub-topics based on the similarity of their target domains, covering aspects from personal challenges to global cooperation. These sub-topics were then further analyzed for their interrelations, and consolidated into 4 broader metaphor topics, each reflecting a distinct sociocultural perspective, providing a structured framework for understanding the role of metaphor in news discourse. The identification of metaphor topics was conducted using the widely applied grounded theory approach (Glaser and Strauss, 1967), employing open coding to integratively categorize target domains based on their significance or universality, forming overarching topics. The coding process was initially carried out independently by three researchers, all adhering to the same set of criteria, followed by cross-checking among the three to ensure consistency in categorization. A final consensus was reached through roundtable discussions. In analyzing metaphor functions, the socio-cultural context of each metaphor theme was first identified, considering current social issues, values, and public concerns. The study then delved into the role of metaphor in communication, particularly how it aids in building social identity, disseminating and educating values, and addressing social issues. Finally, the interplay of metaphor across linguistic, cognitive, and social levels was examined.

## Results and discussion

### Linguistic metaphors and conceptual metaphors

The “from below” linguistic metaphors and “from roundabout” conceptual metaphors together form two essential aspects of metaphor analysis. Given the complexity of metaphorical language and mapping relationships in the Winter Olympics news corpus, analyzing both provides a more comprehensive and in-depth understanding of how metaphors are expressed and cognitively processed in the text, aligning with Cameron’s view that metaphor research must address both language and cognition (Cameron, 2003).

**Figure 2:**
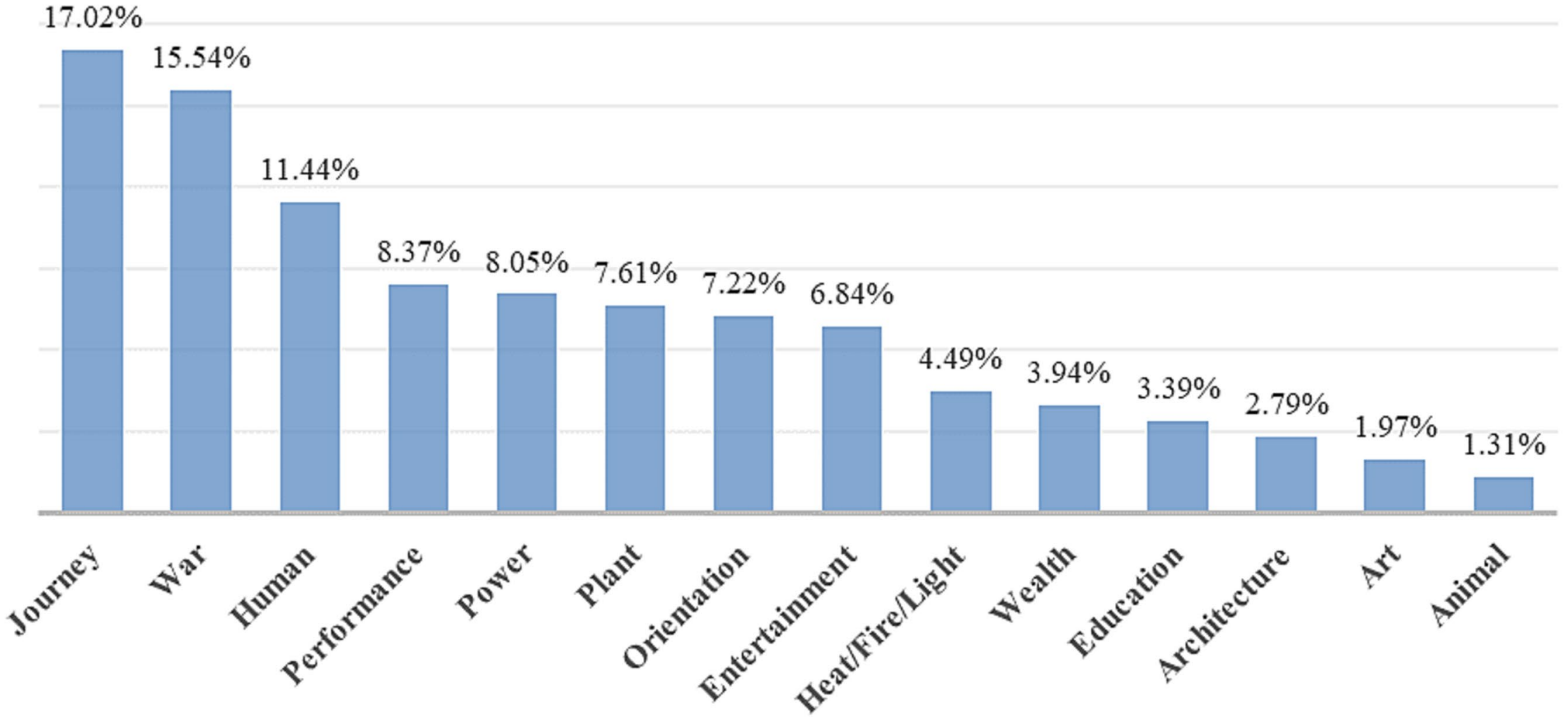
Quantitative distribution of linguistic metaphors in media discourse of Beijing Winter Olympics.

The identification of metaphors in the news corpus revealed a frequency of metaphorical words reaching 1,812 across the three media outlets. A quantitative characterization of metaphorical expressions in media discourse, categorized by source domain, is presented in Figure 2. The results showed a rich variety of metaphorical language in news discourse, which can be grouped into 14 categories based on source domains, with journey (17.02%), war (15.54%), human (11.44%), performance (8.37%), and power (8.05%) being relatively more frequent.

The distribution of linguistic and conceptual metaphors in media discourse, as shown in Table 1, indicated that the conceptual metaphors in Winter Olympics news coverage involve four topics: Olympic spirit (38.96%), values of a community with a shared future for mankind (32.45%), national image of China (21.58%), and the concept of sustainable development (7.01%). Each topic encompassed 2–3 subtopics, which also served as the target domains for the metaphors. These metaphors reflected how the media shaped specific perspectives on the Winter Olympics through metaphorical language. The following discussion will elaborate on linguistic metaphors and their mapping relationships to conceptual metaphors. Due to the abundance of metaphorical sentences, we have chosen some representative examples for illustration. These examples, including the original Chinese versions and their English translations, are placed in the Supplementary material. The translation of metaphorical sentences was jointly deliberated by three researchers.

**Table 1:** Linguistic metaphors and conceptual metaphors in media discourse.

Conceptual metaphors				Linguistic metaphors/high-frequency metaphoric words
Topic	Sub-topic/target domain	Source domain	Frequency	
Olympic spirit (*n* = 706, *p* = 38.96%)	Self-Challenge	War	159	挑战(challenge), 拼搏(fight), 突破(break through)
		Orientation	64	更高(higher), 提升(raise), 高难度(high-difficulty), 顶尖(top)
		Plant	56	收获(reap), 摘(seize)
	Fair Competition	Performance	128	开幕(opening), 舞台(stage), 亮相(appearance), 主角/角色(protagonist), 大幕(act curtain)
		War	125	激战(intense battle), 战胜(overcome), 征战(march into), 战绩(record), 将军(general)
	Unity and Friendship	Journey	107	目标(goal), 飞跃(leap forward), 出发(set off), 迈向(stepping towards), 旅(journey), 起点(start)
		Human	67	携手(hand in hand), 相约(make a date)
Values of a community with a shared future for mankind (*n* = 588, *p* = 32.45%)	Peaceful Development	Journey	163	前行/前进(moving forward), 路(road), 前行之路(road forward), 脚步(step)
		Heat/fire/light	82	照亮(illuminate), 光芒(light), 温暖(warmth)
		Performance	25	奏响(play), 和音(harmony)
	Cultural Exchange	Entertainment	125	盛会(grand gathering), 故事(story)
		Education	62	书写(write), 篇章(chapter), 史册(annals of history)
	Unity and Mutual Assistance	Human	80	共同体(community), 家庭(family), 迎接(welcome)
		Architecture	51	筑(build), 窗口(window), 台阶(level), 平台(platform)
National image of China (*n* = 391, *p* = 21.58%)	Economic Prosperity	Power	147	带动(drive), 动力(driving force), 推动(propel)
		Plant	77	开放(opening up), 成果(fruit), 绽放(bloom)
	Technological Advancement	Journey	41	迈向(step towards), 飞跃(leap forward), 路(road)
		Orientation	38	提升(raise), 高科技(high-tech)
		Human	28	智慧(smart), 智能(intelligent)
	Cultural Splendor	Art	36	雪飞天(Snow Feitian), 底色(underlying color), 画卷(picture)
		Animal	24	雪游龙(snow dragon), 雪飞燕(snow swallow)
Concept of sustainable development (*n* = 127, *p* = 7.01%)	Resource Recycling	Wealth	72	遗产(legacy), 金山银山(mountains of gold and silver)
		Human	19	变身(shapeshift), 华丽转身(graceful turn)
	Environmental Protection	Orientation	30	低碳(low-carbon), 低能耗(low energy consumption)
		Plant	6	生根(taking root), 发芽(sprout)

Note on “Snow Feitian”: It refers to the Shougang Ski Big Jump, which features a design that resonates with the graceful curves of the “Feitian” in Dunhuang murals.

### Olympic spirit

The Olympic spirit is an enduring theme of the Olympic Games. In the media discourse of the Beijing Winter Olympics, the Olympic spirit was imbued with rich symbolic meanings. This sporting event’s core values were communicated through various linguistic and conceptual metaphors. The sub-themes of self-challenge, fair competition, and unity and friendship collectively constructed the dimensions of the Olympic spirit.

### Self-challenge

Self-challenge is a vital component of the Olympic spirit, depicted through different metaphorical perspectives such as war, orientation, and plant metaphors. These metaphors illustrated the inner drive and external performance of athletes in their pursuit of excellence. War metaphors like “challenge,” “fight,” and “break through” portrayed the athletes’ competitive process as a spirited battle, emphasizing their bravery and determination in the face of adversity. Orientation metaphors such as “higher,” “raise,” “high difficulty,” and “top” linked the Olympic spirit to the continuous aspiration for excellence, symbolizing the athletes’ spirit of constantly climbing and striving for higher goals. Plant metaphors like “reap” and “seize” connected the athletes’ efforts to the natural process of growth and harvest, highlighting the process of achieving results in competitions after long periods of training and preparation. These metaphors conveyed the indomitable spirit of the athletes, inspiring both athletes and audiences to pursue higher goals and achieve personal growth and self-fulfillment.

### Fair competition

Fair competition is a core principle of the Olympic spirit, constructed through performance and war metaphors that established elements of open competition at the Winter Olympics, such as the athletic field, athlete confrontation, and overcoming opponents. Performance metaphors like “opening,” “appearance,” “stage,” and “protagonist” likened the sports field to a stage where athletes were the main characters, and their competitions were exciting performances. This metaphor emphasized the importance of athletes showcasing their skills in a fair environment and also implied the audience’s anticipation for thrilling contests and their support for the athletes. War metaphors such as “intense battle,” “overcome,” “march into,” and “record” further underscored the intensity of competition and the athletes’ determination to win fairly. These performance and war metaphors created a passionate and respectful competitive environment where athletes embodied the Olympic spirit of striving and competing.

### Unity and friendship

Unity and friendship are essential values of the Olympic spirit, richly interpreted through journey and human metaphors. Journey metaphors like “goal,” “set off,” and “leap forward” likened an athlete’s competitive career to a journey, emphasizing teamwork and mutual support in the pursuit of objectives. This metaphor conveyed a positive team spirit, with athletes moving forward together under a shared goal, encouraging each other, and facing challenges as one. Human metaphors such as “hand in hand,” and “make a date,” highlighted the close cooperation and support among athletes, reflecting the emotional bonds and mutual understanding between them. These metaphors depicted a scene where athletes supported each other and grew together in the pursuit of excellence, together shaping an Olympic spirit filled with unity, cooperation, and mutual respect.

### Values of a community with a shared future for mankind

The concept of a “community of shared future for mankind” was proposed by Chinese President Xi Jinping, focusing on human development and the future of the world. It is a Chinese idea and solution that presents the ideal of global cooperation and integration through three sub-themes: peaceful development, cultural exchange, and unity and mutual assistance.

### Peaceful development

Peaceful development is the core value of a community with a shared future for mankind, vividly illustrated through journey, heat/fire/light, and performance metaphors. The journey metaphor, using terms like “moving forward,” “road,” and “steps” likened peaceful development to an ever-extending road, symbolizing the collective efforts of nations in advancing the global economy and society. Heat/fire/light metaphors, with words such as “illuminate,” “light,” and “warmth” emphasized the bright prospects and comforting strength brought by peaceful development, offering hope and opportunities to all countries. The performance metaphor used “play” and “harmony” to symbolize the harmonious cooperation and integration of the world’s countries, stressing the vision of seeking common ground while reserving differences and co-creating a harmonious world. These metaphors presented a beautiful vision of global peaceful development and shared prosperity.

### Cultural exchange

The cultural exchange acts as a bridge and bond within a community with a shared future for mankind, vividly portrayed through entertainment and education metaphors. Entertainment metaphors like “grand gathering,” and “story” likened cultural exchange to a grand celebration, emphasizing its pleasure and appeal, conveying that cultural exchange was not only the transmission of knowledge but also an emotional resonance and spiritual enjoyment. Education metaphors like “write,” “chapter,” and “annals of history” compared cultural exchange to a learning and educational process, highlighting its role in the inheritance of knowledge and the accumulation of wisdom. Through these metaphors, the sub-theme of cultural exchange was endowed with profound connotations; it was not only a display and appreciation of culture but also a transmission of knowledge and an enlightenment of wisdom.

### Unity and mutual assistance

Unity and mutual assistance are indispensable spirits within a community with a shared future for mankind, deeply reflected through human and architecture metaphors. Human metaphors such as “community,” “family,” and “welcome” likened the global population to one big family, with people of all countries united and supportive with each other, conveying a sense of warmth and belonging akin to family. Architecture metaphors compared unity and mutual assistance to the foundation and process of building, emphasizing their foundational role in constructing a stable and prosperous society. Metaphors like “build” illustrated that unity and mutual assistance formed the solid base for a harmonious society, while “window” and “platform” symbolized the pathways provided for mutual understanding and progress among people around the world.

### National image of China

National image is a visualization of a country’s comprehensive strength and plays a strategically vital role in international competition and cooperation. China has used the Winter Olympics as a significant window to showcase its national image, highlighting its achievements and influence in various fields through the sub-themes of economic prosperity, technological advancement, and cultural splendor.

### Economic prosperity

Economic prosperity is a crucial aspect of China’s national image, represented through metaphors of power and plant. Power metaphors such as “drive,” “drive force,” and “propel” likened China’s economic vitality to a powerful force, emphasizing its robust growth and contribution to global expansion. Plant metaphors compared China’s economic boom to the natural and orderly growth of plants. The metaphor of “opening up” conveyed the openness of China’s economy and its widening participation in the international economic system. Terms like “fruit” and “bloom” vividly described the notable successes of China’s economy. These metaphors not only depicted the prosperity of China’s economy but also demonstrated its significant position and positive contributions in the era of globalization.

### Technological advancement

Technological progress is another highlight of China’s national image, showcased through journey, orientation, and human metaphors. Journey metaphors expressed China’s commitment to continuous exploration and breakthroughs in the field of technology. Phrases like “step towards” described the rapid development and innovation capabilities of Chinese technology, while “leap forward” metaphors underscored the role of technological progress in propelling the country’s overall development. Orientation metaphors like “raise” suggested the continuous improvement of China’s technological standards, with “high-tech” directly indicating the internationally advanced levels China has reached in certain tech fields. Human metaphors attributed the ingenuity behind technological innovation to human intelligence, with “smart” and “intelligence” pointing to the development of frontier technologies like artificial intelligence. These metaphors endowed China’s advancements in technology with more humanized and intelligent qualities, showing a harmonious progression of technology and human development.

### Cultural splendor

Cultural splendor is another core element of China’s national image, depicted in media discourse through art and animal metaphors. Art metaphors, such as “Snow Feitian,” captured the agility and elegance of Chinese culture, while “underlying color” and “picture” metaphors emphasized their foundational and profound influence on world culture. Animal metaphors like “snow dragon” evoked the powerful and mysterious image of the dragon in Chinese culture, symbolizing its vitality and strength. “Snow swallow” linked the light and graceful image of the swallow in Chinese culture with the winter sports of the Olympics, reflecting the softness and agility of Chinese culture. Through these imaginative metaphors, media discourse conveyed the unique charm of Chinese culture and its significant position in global culture.

### Concept of sustainable development

The concept of sustainable development refers to growth that meets the needs of the present without compromising the ability of future generations to meet their own needs. In China, environmental protection has become an essential component of national strategy, with the government implementing a series of measures to protect and improve the environment. In the media discourse of the Beijing Winter Olympics, the idea of sustainable development was showcased through the sub-themes of resource recycling and environmental protection, highlighting China’s efforts and achievements in promoting green growth and ecological civilization.

### Resource recycling

As a core concept of sustainable development, resource recycling was expressed through wealth and human metaphors. The wealth metaphor “legacy” emphasized the importance of resource recycling for future generations, signifying not just current wealth but also responsibility for what is to come. The phrase “mountains of gold and silver” vividly described the dual economic and social benefits of resource recycling, reflecting the long-term interests brought by green development. Human metaphors likened resource recycling to humanity’s transformation and upgrading in the face of environmental challenges, underscoring our central role in the recycling process. Metaphors like “shapeshift” and “graceful turn” illustrated innovations in resource management and utilization, as well as proactive responses and changes to environmental issues. Such metaphors encouraged more sustainable and eco-friendly resource use, thereby achieving sustainable development in human society.

### Environmental protection

Environmental protection, a key area of sustainable development, was represented through orientation and plant metaphors. Orientation metaphors likened environmental protection to a direction and goal, emphasizing the importance of reducing carbon emissions and energy consumption. “Low-carbon” and “low energy consumption” represented a shift in production and lifestyle, as well as the construction of a society friendly to the environment. Plant metaphors compared environmental protection to the growth process of plants, highlighting the natural resilience and vitality of the environment and the concept of sustainable development through environmental conservation. Metaphors like “taking root” and “sprout” conveyed the foundational and long-term nature of environmental protection efforts, which requires ongoing commitment and meticulous care to ensure the health of the environment and ecological balance.

In summary, a comprehensive analysis can be conducted on both the “from below” linguistic metaphors and the “from roundabout” conceptual metaphors. From the linguistic perspective, metaphors effectively concretize abstract concepts through careful word choice, skillful sentence structures, and vivid rhetoric, thereby enhancing the expressiveness and appeal of language. For instance, likening athletes to “generals” not only shapes them as heroic figures but also reinforces the conveyance of the Olympic spirit. Comparative and parallel sentence structures emphasize the similarities in athletes’ skills and the diversity of the Winter Olympics, while rhetorical devices such as exaggeration and personification liken the Games to a dramatic “opening” and “stage,” highlighting its status as a global event. Additionally, the density of metaphors in the text plays a crucial role; the intensive use of war metaphors, for example, significantly intensifies the fierceness of the competition. These linguistic metaphors not only convey rich information but also deepen the reader’s understanding of the subject matter, reflecting the media’s strategies and skills in news report writing.

From the conceptual standpoint, metaphors map core concepts such as the Olympic spirit, the values of a community with a shared future for mankind, China’s national image, and the concept of sustainable development. The Olympic spirit is mapped through metaphorical sources like war, journey, and plant, reflecting the athletes’ self-challenge, fair competition, and unity and friendship, thus reinforcing the centrality of sportsmanship at the Winter Olympics. The values of a community with a shared future are mapped through journey and education metaphors, promoting ideals of peaceful development and mutual assistance, and fostering a global civic consciousness. China’s national image is mapped through metaphors of power and journey, portraying the nation’s prosperity and progress, and enhancing public national identity. The concept of sustainable development, through metaphors of wealth and human, is mapped to resource recycling and environmental protection, raising public awareness of environmental issues and encouraging a shift towards environmentally friendly production and lifestyle. These conceptual metaphors enrich the public’s emotional experience at the cognitive level, promoting a deeper understanding and positive action towards important concepts.

### The social function of metaphor

The “from above” perspective focuses on the social attributes of conceptual metaphors, specifically how metaphors achieve communicative purposes within particular social contexts and influence and shape public cognition and attitudes towards the Winter Olympics. This analysis synthesizes the news discourse of the Winter Olympics and the literature research findings, providing an interpretive framework for understanding the social functions of metaphors. While our examination does not involve direct empirical verification through surveys or experiments, it provides a conceptual framework ripe for future empirical exploration.

### Building social identity and unity

In the reporting of the Beijing Winter Olympics, metaphors are poised to play a pivotal role in constructing social identity and unity. By comparing athletes’ endeavors to wars and journeys, metaphors have the potential to deepen the public’s resonance with the Olympic spirit and strengthen a collective sense of identity. The employment of such metaphors is, in effect, shaping and reinforcing a collective memory that is closely connected to the nation’s historical, cultural, and value-laden narratives (Laikhuram, 2023; Zubrzycki and Woźny, 2020). These metaphors evoke recollections of heroic struggles integral to the nation’s history, thereby fortifying a sense of national pride and collective cohesion. Furthermore, metaphors can act as a bridge in cross-cultural communication. Notably, the conceptual metaphor of a “community with a shared future for mankind” offers a shared framework that fosters mutual understanding and unity among different cultures. It accentuates the universal aspects of various cultures, aiding in the dissolution of cultural divides and nurturing a sense of global citizenship (Khan et al., 2021). This, in turn, propels the establishment of multi-tiered social identities, which is instrumental in fostering societal harmony.

### Promoting the dissemination and education of values

The metaphors used in the reporting of the Winter Olympics will become an important means of conveying and educating social values. By linking the conceptual metaphor of “a community with a shared future for mankind” with “peaceful development” and “cultural exchange,” the media communicated the ideals of global cooperation and integration, educating the public to understand and embrace these values. The use of metaphors helped to foster a common pursuit of peace, development, and cultural diversity, advancing the spread and education of values at the sociocultural level (Kövecses, 2003). Moreover, metaphors carried deep cultural meanings and social orientations, playing a significant role in shaping social values. Through metaphors of “fair competition” and “unity and friendship,” media discourse conveyed values of respect for opponents, pursuit of excellence, and international friendship, transforming the abstract Olympic spirit into concrete emotional experiences that influenced public values. Additionally, metaphors, with their capacity to encapsulate complex ideas and emotions, are likely to serve as conduits for new perspectives on value education (Jensen, 2006). For instance, by employing innovative metaphors that resonate with contemporary issues such as environmental sustainability, the media can introduce and emphasize values that are relevant to the challenges and opportunities of the modern era.

### Promoting focus on and resolution of social issues

Metaphors play a significant role in drawing attention to and resolving social issues by concretizing abstract societal concepts, enabling the public to intuitively feel the urgency and necessity of addressing these problems (Musolff, 2006). In the coverage of the Beijing Winter Olympics, the use of metaphors will increase public awareness of global environmental issues and deepen the understanding of the importance of sustainable development. For instance, the metaphor of “resource recycling” emphasized the importance of reusing resources, while the metaphor of “environmental protection” highlighted the urgency of preserving natural ecosystems. These metaphors may raise public consciousness about environmental issues and inspire social attention and action towards sustainable development (Princen, 2010). Additionally, metaphors of fair competition stressed the importance of equal opportunities and fair treatment for all athletes in sports events, helping the public recognize the broader significance of fair competition in society. Metaphors of unity and friendship, on the other hand, underscored the need for people from different countries and cultural backgrounds to cooperate in facing common challenges.

### Facilitating social change and reality construction

By challenging and reshaping public cognitive frameworks and introducing new perspectives and ideas, metaphors will promote the renewal and transformation of social concepts (Charteris-Black, 2004). In the reporting on the Beijing Winter Olympics, the media skillfully used metaphors to associate China’s national image with concepts such as plant growth and the process of journey, showcasing China’s active stance and contributions to the process of globalization, which will help refresh the international community’s traditional views of China and foster the harmonious development of international relations. At the same time, as builders of social reality, metaphors effectively linked abstract ideas with concrete practices (Gutiérrez Escalante, 2019). By combining metaphors of China’s national image with concepts such as economic prosperity, technological advancement, and cultural splendor, the media have shaped a positive national image, which will enhance the public’s awareness and pride in the country’s developmental achievements. Thus, conceptual metaphors, by providing new cognitive frameworks, participated in the construction of social reality, laying a solid cultural and psychological foundation for social change.

The social functions reflected by these metaphors are deeply intertwined with the cultural and historical context of contemporary China, particularly in promoting the Olympic spirit, fostering a harmonious society, and crafting an image of a green, open, and innovative nation (Han and Zhang, 2018; Hu and Zhao, 2013). They encapsulate China’s proactive efforts to project a positive image on the global stage, reflecting its commitment to sustainable development, technological advancement, and cultural prosperity. Through these metaphors, we discern how China leverages sporting events to convey its core values and developmental philosophy, thereby establishing a proactive, collaborative, and responsible national persona in the international community.

### The interplay of language, cognition, and social functions of metaphors

The analysis of metaphors in the news discourse of the Beijing Winter Olympics under the “Trinocular Perspective” demonstrates the diversity and complexity of metaphors. It reveals that the language, cognition, and social functions of metaphors are interwoven, and this section will integrate these analyses and incorporate literature perspectives to expose how metaphors interact across linguistic, cognitive, and social functional levels.

### The interplay of language and cognition

The linguistic expression of metaphors is closely linked to their cognitive functions. Language transforms abstract concepts into tangible images through carefully selected vocabulary, skillful sentence structures, and vivid rhetorical devices, allowing people to intuitively perceive and understand these concepts (Lupyan, 2016). For example, likening the Beijing Winter Olympics to “a new chapter in history” not only imbues the event with meaning and depth linguistically but also facilitates cognitive understanding of the Olympics’ place in the historical process. Conversely, cognition influences language as individuals select and create linguistic expressions through internal thought patterns and conceptual understanding (Li et al., 2024). Cognitive frameworks guide linguistic innovation and diversity, enabling language to adapt to evolving cognitive demands and communication purposes (Zawada, 2006). For instance, the metaphor of technological progress as a “journey” understands the development of technology as a continuous process of exploration and advancement. This internal thought pattern drives linguistic innovation, depicting technological progress as a journey filled with discoveries. The combination of language and cognition makes metaphor a powerful cognitive tool, aiding in the construction and activation of relevant conceptual frameworks in the brain, thus promoting the cognitive processing and understanding of complex concepts (Myachykov et al., 2013).

### The interplay of cognition and social function

The cognitive role of metaphors is intertwined with their impact on social functions. Metaphors enable understanding and acceptance of abstract concepts within sociocultural contexts through mapping processes (Landau et al., 2010). For example, the metaphor “a community with a shared future for mankind” not only aids cognitive understanding of interdependence in the era of globalization but also promotes the concept of international cooperation and shared development in social functions, facilitating the spread and education of values at the sociocultural level. The influence of social functions on cognition is seen in how sociocultural backgrounds and values shape individual cognitive frameworks (Wascher et al., 2018). Social behaviors and public policies, through the dissemination and practice of metaphors, deepen understanding and identification of specific concepts, and foster the formation of new cognitive patterns and shifts in cognitive paradigms (Burgers, 2016). For instance, by comparing environmental protection to a precious “legacy,” public awareness of sustainable development is enhanced, and advocacy for such social behavior promotes a new cognitive model of the importance of environmental protection, influencing people’s recognition and support for environmental policies. The interplay between cognition and social function concretizes abstract social values, making them easier for the public to understand and accept, and guiding social behavior and decision-making.

### The interplay of language and social function

The use of metaphors at the linguistic level interacts with their effects on social functions. Specific metaphors chosen not only convey information but also influence social attitudes and behaviors. In news reporting, the media can shape public perceptions and emotional responses to events by employing particular linguistic metaphors (Landau and Keefer, 2014; Thibodeau et al., 2019). For example, describing economic development as a “blooming flower” linguistically creates a positive image and socially inspires confidence and anticipation for the future. The impact of social functions on language is reflected in how societal needs and values drive the development and evolution of language. Social events and collective actions prompt the creation of new metaphors and the progression of language to adapt to changing communication purposes and societal expectations (Akay, 2007; Saha, 2019). For instance, portraying China’s economy through the power metaphor as a driver of global growth mirrors society’s positive evaluation of the country’s developmental vitality, demonstrating how social values guide linguistic expression. Through such metaphors, language evolves to meet society’s needs to shape and convey national identity. This interplay between language and social functions can influence people’s thoughts and actions through the power of language, shaping the sociocultural environment and fostering social unity and progress.

**Figure 3:**
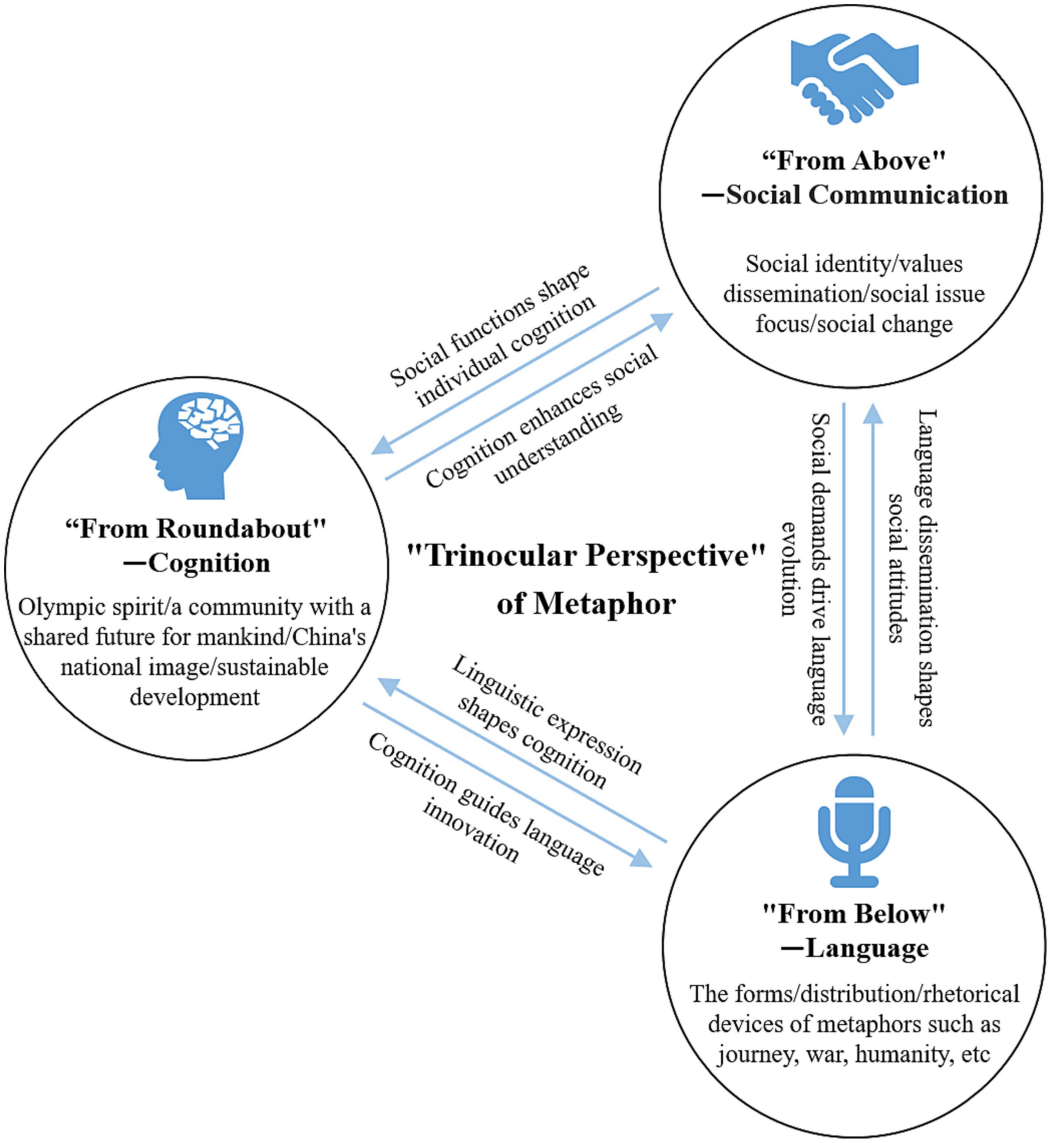
Interplay model of language, cognition, and social functions of metaphor.

In summary, the linguistic, cognitive, and social functional levels of metaphors interact with each other, forming a dynamic and mutually shaping complex system (Figure 3). Within this system, metaphorical linguistic expressions enrich cognitive frameworks and facilitate understanding of complex concepts; cognitive frameworks, through multi-themed conceptual mappings, guide the internalization and dissemination of social values; and social functions, based on sociocultural contexts, influence the strategic use of metaphorical language to guide public cognition, thereby achieving communicative purposes and shaping social identity and values. This dynamic system not only enhances the effectiveness of information transmission but also deepens the public’s understanding and emotional resonance with the Beijing Winter Olympics and the deeper values it represents. Through metaphors, we can communicate and understand multifaceted social phenomena and continuously shape and reshape our social reality in communication, reflecting the profound impact of metaphors at linguistic, cognitive, and social functional levels.

## Conclusion

This research utilized the “Trinocular Perspective” framework to investigate the multifaceted roles of metaphor in news coverage of the Beijing Winter Olympics. The findings demonstrated that metaphors not only enrich the linguistic landscape of news reporting but also serve as powerful cognitive and social tools. They encapsulate the essence of the Olympic spirit and a community with a shared future for mankind, reflect on China’s evolving global image, and underscore the urgency of sustainable development, thereby constructing social identity, disseminating values, and promoting social progress. The study further revealed that the linguistic, cognitive, and social functional dimensions of metaphor interact to create a dynamic, interdependent complex system, reinforcing the multiple roles of metaphor in shaping discourse, molding public cognition, and guiding social values.

This study’s significance lies in its theoretical and methodological advancements in understanding the intricate roles of metaphors in shaping media discourse, influencing public cognition, and guiding social values, particularly within the context of the international mega-events. However, it also acknowledges the limitations inherent in its approach; the exploration of the social functions of metaphors and the interplay among language, cognition, and social dimensions are based on corpus analysis and literature synthesis, without empirical validation through experiments or surveys. Nevertheless, this research offers a robust foundation for future empirical inquiries and practical applications in the fields of linguistics and journalism. Future research should extend the “Trinocular Perspective” to cross-cultural sports communication, examining how metaphors influence cognitive patterns and social behaviors, and their strategic use in enhancing international understanding and cooperation within the sports media landscape.

## Data Availability

The original contributions presented in the study are included in the article/Supplementary material, further inquiries can be directed to the corresponding author.
