# Supplementary material for: Metaphorical discourse in Beijing Winter Olympic news: a Trinocular Perspective analysis of language, cognition, and social functions
Source: Front Psychol. 2024 Dec 17;15:1477890. doi: 10.3389/fpsyg.2024.1477890 (PMC11685200; doi:10.3389/fpsyg.2024.1477890)
Supplement: Supplementary file 1 [file Data_Sheet_1.pdf]

Supplementary Material

Table S1 Examples of metaphorical sentences and English translations of the Olympic spirit

Sub-topic	Metaphorical Sentences	English Translations	Source
Self-Challenge	赛场成为她**挑战**自我的舞台，反脚 1260 度动作、连续 1080 度转体动作引起阵阵惊呼。	The arena transformed into her stage for self-**challenge**, where her counter-rotation 1260-degree moves and consecutive 1080-degree spins sparked rounds of amazement.	People’s Daily, 2022/2/14
	面对挫折，咬牙坚持，**拼搏**到底，是许多运动员的共同选择。	Facing setbacks, many athletes choose to grit their teeth and **fight** to the end.	Xinhua Net, 2022/2/19
	从 12 年前第一次参加冬奥会，到 4 年前第一次站上冬奥会领奖台，4 次征战冬奥会的刘佳宇一路披荆斩棘、**突破**自己。	From her first Winter Olympics 12 years ago to her first time on the podium 4 years back, Liu Jiayu, a four-time Winter Olympics competitor, has consistently overcome obstacles and **broken through** her personal barriers.	People’s Daily, 2022/2/14
	现代奥林匹克运动作为全世界最高级别的体育盛会，“更快、**更高**、更强”当然是奥林匹克的重要宗旨。	The modern Olympic Games, as the premier sporting event worldwide, naturally embrace the important tenet of "Faster, **Higher**, Stronger."	China Daily，2022/2/9
	从第二跳开始，瑞士队和美国队选手纷纷**提升**难度，让单跳分数屡创新高。	Starting with the second jump, Swiss and American competitors **raised** the difficulty, causing the scores for individual jumps to reach new heights repeatedly.	People’s Daily，2022/2/11
	近年来，挑战**高难度**动作成为花样滑冰特别是单人滑项目的趋势之一。	In recent years, taking on **high-difficulty** maneuvers has become one of the trends in figure skating, especially in the singles discipline.	People’s Daily, 2022/2/14

	**顶尖**高手之间荡气回肠的彼此激发、尊重与共情，这就是体育精神。	The soul-stirring mutual inspiration, respect, and empathy among **top** athletes is the essence of sportsmanship.	Xinhua Net，2022/2/10
	本届冬奥会上，41 岁的老将克拉雷**收获**了 4 届冬奥之旅的第一枚奖牌。	At this Winter Olympics, Clare, the 41-year-old veteran, **reaped** her first medal from four Olympic journeys.	People’s Daily, 2022/2/8
	当韩国选手黄大宪**摘**得桂冠后，中国网友也积极称赞，“谁赢了都是奥运英雄”	After South Korean athlete Hwang Dae-Heon **seized** the championship, Chinese netizens also actively praised, "Whoever wins is a hero of the Olympics."	Xinhua Net, 2022/2/10
Fair Competition	北京冬奥会**开幕**以来，各项赛事精彩纷呈，收视率在欧美多国成倍增长，甚至打破纪录。	Since the **opening** of the Beijing Winter Olympics, the competitions have been thrilling, with viewership doubling and even breaking records in many European and American countries.	China Daily, 2022/2/16
	北京 2022 年冬奥会为全世界的运动员提供了一个公平竞技的**舞台**。	The Beijing 2022 Winter Olympics provided a **stage** for athletes from around the world to compete fairly.	China Daily，2022/2/10
	运动员是奥运会的**主角**，奥运会对许多运动员来说也许是一生只有一次的机会。	Athletes are the **protagonists** of the Olympic Games, an opportunity that may come once in a lifetime for many.	People’s Daily，2022/2/5
	在北京冬奥会女子单人雪车比赛中，怀明明和应清完成了中国女子雪车在冬奥会赛场上的首次**亮相**。	In the women's singles bobsled competition at the Beijing Winter Olympics, Huai Mingming and Ying Qing made their first **appearance** for China's female bobsledding at the Winter Olympics.	People’s Daily, 2022/2/15
	北京 2022 年冬奥赛场**激战**正酣，赛事热度不减。	The **intense battles** of the 2022 Beijing Winter Olympics are in full swing, with the heat of the competition showing no signs of letting up.	China Daily，2022/2/8
	最终瑞士选手马尔科·奥德马特**战胜**了对手，也“战胜”了天气，以两轮 2 分 09 秒 35 的总成绩获得金牌。	Ultimately, Swiss athlete Marco Odermatt **overcame** his rivals and, metaphorically, the weather, securing the gold medal with a total time	Xinhua Net, 2022/2/13

		of 2:09.35 over two runs.	
	当速度滑冰成为她的追求，没有什么可以阻挡她**征战**冬奥会的脚步。	When speed skating became her pursuit, nothing could stop her from **marching into** the Winter Olympics.	People’s Daily, 2022/2/7
	在 2 月 8 日的冰壶混双决赛中，意大利队以不败**战绩**首次获得金牌。	In the curling mixed doubles final on February 8th, the Italian team secured their first gold medal with an unblemished **record**.	People’s Daily，2022/2/17
	中国短道速滑队 5 日惊险夺得北京冬奥会短道速滑混合团体接力金牌后，接力团队**众将**不约而同地将夺冠的关键归于“团队的力量”。	After the Chinese short track speed skating team narrowly won the gold medal in the mixed relay at the Beijing Winter Olympics on the 5th, the relay team's **generals** all attributed the key to winning the championship to the "strength of the team."	Xinhua Net，2022/2/6
Unity and Friendship	北京冬奥会口号“一起向未来”体现了奥林匹克运动的核心价值观和愿景，以及追求世界团结、和平与进步的**目标**。	The slogan of the Beijing Winter Olympics, "Together for a Shared Future," embodies the core values and vision of the Olympic Movement, as well as the **goal** of pursuing world unity, peace, and progress.	People’s Daily，2022/2/10
	对她和这支年轻的中国队而言，踏上北京冬奥的赛场，就是站在新起点上的一次**飞跃**。	For her and this young Chinese team, stepping onto the Beijing Winter Olympics' field is a **leap forward** at a new starting point.	Xinhua Net, 2022/2/7
	表达世界大爱与和平的音乐主题，展现北京冬奥会无穷力量和伟大精神，从冬奥**出发**，**迈向**世界。	The musical theme expressing universal love and peace demonstrates the endless strength and great spirit of the Beijing Winter Olympics, **setting off** from the Winter Olympics and **stepping towards** the world.	China Daily, 2022/2/9
	北京冬奥会如期举行让运动员能够完成圆梦之**旅**。	The Beijing Winter Olympics were held as scheduled, allowing athletes to complete the **journey** of fulfilling their dreams.	People’s Daily, 2022/2/5

	卡普拉洛斯指出，北京冬奥会将继续展示奥林匹克精神带领人们**携手**走出困境的决心。	Kapralos pointed out that the Beijing Winter Olympics will continue to demonstrate the determination of the Olympic spirit to lead people out of difficulties **hand in hand**.	People’s Daily, 2022/2/7
	节目《相亲相爱》用各国冬奥健儿互相鼓励的画面诠释“相互理解、友谊团结、公平竞争”的奥林匹克精神，传递了“友谊长存，**相约**未来”的美好愿望。	The program "Xiang Qin Xiang Ai" interprets the Olympic spirit of "mutual understanding, friendship solidarity, and fair competition" with scenes of winter athletes from various countries encouraging each other, conveying the beautiful wish of "eternal friendship, **making a date** in the future."	China Daily，2022/2/19

Table S2 Examples of metaphorical sentences and English translations of the values of a community with a shared future for mankind

Sub-topic	Metaphorical Sentences	English Translations	Source
Peaceful Development	北京冬奥会开幕式体现了人性的光辉，也展现了各国人民团结**向前**的人类命运共同体理念和奥林匹克精神。	The opening ceremony of the Beijing Winter Olympics embodied the brilliance of humanity and also demonstrated the concept of a community with a shared future for mankind and the Olympic spirit, with people from all countries uniting and **moving forward.**	People's Daily, 2022/2/6
	生生不息的奥运之火，辉映"双奥之城"的光荣与梦想，照耀人类共克时艰、团结合作的**前行之路**。	The ever-burning Olympic flame reflects the glory and dreams of the "dual Olympics city," illuminating the **road forward** for humanity to overcome difficulties together and unite in cooperation.	Xinhua Net, 2022/2/4
	中国有越来越多孩子学习并参与冰雪运动，很像人类前进的**脚步**——跌倒了爬起来，再跌倒再爬起来，快乐地运动，再继续向前。	More and more children in China are learning and participating in ice and snow sports, much like the **steps** of human progress – falling and getting up, falling again and getting up again, exercising happily, and continuing to move forward.	People's Daily, 2022/2/6
	缓缓上升的"大雪花"，依然散发着耀眼的**光芒**，**照亮**每个人努力奋进、追逐梦想的人生之路，照亮全人类携手前行、共克时艰的美好明天。	The slowly rising "big snowflake" still emits a dazzling **light**, **illuminating** the path of life for everyone striving and pursuing dreams, and lighting up the beautiful tomorrow where all of humanity moves forward together to overcome difficulties.	Xinhua Net, 2022/2/4
	这场举世瞩目的体育盛会，不仅为身处疫情寒冬的各国人民带来了**温暖**与希望，更为动荡不安的世界注入了和平与团结的宝贵力量。	This world-famous sports event not only brought **warmth** and hope to people in various countries during the cold winter of the pandemic but also injected valuable strength of peace and unity into a turbulent world.	China Daily, 2022/2/19

	中华文明与奥林匹克运动再度携手，**<u>奏响</u>**全人类团结、和平、友谊的华美乐章。	Chinese civilization and the Olympic Movement join hands again to **<u>play</u>** the magnificent movement of unity, peace, and friendship for all mankind.	People's Daily, 2022/2/6
	在不同地方，通过不同方式，用不同语言，冬奥志愿者向各国各地区运动员和嘉宾展示友好，传递热情，唱响"一起向未来"的**<u>和音</u>**。	In different places, through different means, and in different languages, Winter Olympics volunteers show friendliness to athletes and guests from all countries and regions, convey enthusiasm, and sing the **<u>harmony</u>** of "together towards the future."	People's Daily, 2022/2/11
Cultural Exchange	通过电视转播镜头，全球观众看到了一个团结、励志、拼搏、温情、和谐的冬奥**<u>盛会</u>**，这正是全人类共同期盼的图景，也是"更团结"的现实写照。	Through the television broadcast lens, global audiences witnessed a united, inspiring, striving, warm, and harmonious Winter Olympic **<u>grand gathering</u>**.	Xinhua Net, 2022/2/12
	当奥林匹克之火再度照亮北京的夜空，照亮人们的心头，一段关于拼搏与梦想、团结与和平的**<u>故事</u>**将从这里起笔。	As the Olympic flame once again illuminates Beijing's night sky and people's hearts, a **<u>story</u>** about striving and dreams, unity and peace will begin to be written here.	People's Daily, 2022/2/5
	北京冬奥会**<u>书写</u>**了国际体育史上的新**<u>篇章</u>**，中国为奥林匹克运动和推动构建人类命运共同体作出了重要贡献。	The Beijing Winter Olympics has **<u>written</u>** a new **<u>chapter</u>** in the history of international sports.	People's Daily, 2022/2/12
	北京冬奥会一定会成为简约、安全、精彩的奥运会而载入**<u>史册</u>**。	The Beijing Winter Olympics will surely be recorded in the **<u>annals of history</u>** as a simple, safe, and splendid Olympic Games.	Xinhua Net, 2022/2/7
	习近平主席以"诺亚方舟"为喻，传递中国推动构建人类命运**<u>共同体</u>**的决心	President Xi Jinping uses the metaphor of "Noah's Ark" to convey China's determination to promote the construction of a **<u>community</u>** with a shared future for mankind.	Xinhua Net, 2022/2/7

Unity and Mutual Assistance	我们应该弘扬奥林匹克运动精神，团结应对国际社会共同挑战，践行真正的多边主义，共同建设和谐合作的国际大**家庭**。	We should promote the spirit of the Olympic Movement, unite to face the common challenges of the international community, practice true multilateralism, and jointly build a harmonious and cooperative international **family**.	People’s Daily, 2022/2/6
	以《立春》为题的表演极具巧思，绿色的充满希望和活力的演绎方式点燃全场，寓意着世界人民共同**迎接**一个新的春天。	The performance titled "Beginning of Spring" is full of ingenuity, the green interpretation full of hope and vitality ignites the whole venue, symbolizing that the people of the world are **welcoming** a new spring together.	China Daily, 2022/2/4
	**筑**梦冰雪，同向未来。	**Building** dreams on ice and snow, heading towards the future together.	China Daily, 2022/2/8
	冬奥会体育展示是衔接竞赛与观众之间的纽带，是传递奥林匹克精神、展示主办国文化的重要**窗口**。	The Winter Olympics sports presentation is the link between competition and spectators, an important **window** for conveying the Olympic spirit and showcasing the host country's culture.	China Daily, 2022/2/16
	中国在冬季运动方面取得的非凡成就，开启了全球冬季运动的新时代，将使全球冬季运动参与度登上新**台阶**，让中国人民和全球各地的冬季运动爱好者从中受益。	China's extraordinary achievements in winter sports have ushered in a new era of global winter sports, which will bring the global participation in winter sports to a new **level,** benefiting the Chinese people and winter sports enthusiasts around the world.	People’s Daily, 2022/2/5
	作为一场世界瞩目的奥运赛事，约 90 个国家和地区的近 3000 名冰雪健儿共赴冰雪之约，为促进世界和平、实现文化交流、传递友谊情感搭建了交流**平台**。	As an Olympic event watched by the world, nearly 3,000 ice and snow athletes from about 90 countries and regions have come together for the ice and snow rendezvous, building a **platform** for promoting world peace, achieving cultural exchange, and conveying the sentiment of friendship.	China Daily, 2022/2/10

Table S3 Examples of metaphorical sentences and English translations of the national image of China

Sub-topic	Metaphorical Sentences	English Translations	Source
Economic Prosperity	京津冀协同发展，通过发展冰雪产业**带动**区域脱贫振兴，解决发展不平衡的问题。	The coordinated development of the Beijing-Tianjin-Hebei region, through the development of the ice and snow industry, **drives** regional poverty alleviation and revitalization, addressing the issue of unbalanced development.	People’s Daily, 2022/2/4
	北京冬奥会体现了区域一体化发展理念，是中国经济高质量发展的重要**动力**。	The Beijing Winter Olympics embodies the concept of regional integrated development and is an important **driving force** for high-quality economic development in China.	People’s Daily, 2022/2/14
	随着投资增加和基础设施的完善，张家口坚持生态优先，**推动**绿色发展，经济发展获得新动力，成为中国冬季运动产业的聚集地和旅游胜地。	With increased investment and improved infrastructure, Zhangjiakou adheres to ecological priority and **propels** green development, gaining new momentum for economic growth and becoming a hub for China's winter sports industry and a popular tourist destination.	People’s Daily, 2022/2/14
	在两国元首引领下，哈中在政治、安全、经贸、人文等领域的合作取得丰硕**成果**，“光明之路”新经济政策与共建“一带一路”倡议的对接开辟了广阔的合作前景。	Under the guidance of the leaders of the two countries, Kazakhstan and China have borne rich **fruits** in cooperation across political, security, economic, and cultural fields. The alignment of Kazakhstan's "Bright Road" new economic policy with the joint "Belt and Road" initiative opens up broad prospects for cooperation.	People’s Daily, 2022/2/9
	“今天的中国跟 2008 年时的中国又不一样，深化改革**开放**让中国取得了巨大成果，实现脱贫攻坚胜利后的人民精神面貌焕然一新。	Today's China is different from China in 2008; deepening reform and **opening up** has brought about significant achievements, and the	Xinhua Net, 2022/2/4

		people's spirit has been rejuvenated after the victory in poverty alleviation.	
	中国在世界舞台上**绽放**精彩，成为世界经济发展的重要力量。	China has **bloomed** on the world stage and become an important force in world economic development.	People’s Daily, 2022/2/10
Technological Advancement	北京冬奥会不仅展现了中国的经济发展、科技实力和国际影响力，也标志着中国正在向体育强国不断**迈进**。	The Beijing Winter Olympics not only showcases China's economic development, technological strength, and international influence but also signifies that China is continuously **stepping towards** becoming a powerful sports nation.	People’s Daily, 2022/2/5
	为了帮助运动员在北京冬奥会上取得最佳成绩，“冰丝带”首次采用了二氧化碳跨临界直冷制冰技术，这是一项技术上的**飞跃**。	To help athletes achieve their best results at the Beijing Winter Olympics, the "Ice Ribbon" has adopted carbon dioxide transcritical direct cooling ice-making technology for the first time, marking a technological **leap forward**.	China Daily，2022/2/17
	同我们国家的强国之路一样，中国冰雪运动也必须走科技创新之**路**。	Just like the path to becoming a powerful nation for our country, China's ice and snow sports must also follow the **road** of technological innovation.	People’s Daily, 2022/2/4
	从东京夏季奥运会到北京冬季奥运会，国际大型赛事中的智能技术含量在过去半年中的**提升**明显，中国的高新技术实力也在本届冬奥会上得到充分体现。	From the Tokyo Summer Olympics to the Beijing Winter Olympics, the content of intelligent technology in international large-scale events has significantly **raised** in the past six months, and China's high-tech strength has also been fully demonstrated at this Winter Olympics.	China Daily, 2022/2/4
	正因中国在广播和电信技术、工业方面如此成熟，我们才有机会使用这些**高科技**。	It is precisely because China is so mature in broadcasting, telecommunications technology, and industry that we have the opportunity to use this **high technology**.	Xinhua Net, 2022/2/11

	北京冬奥会掀起冰雪运动的风尚潮流，中国高铁以<u>智慧</u>升级、<u>智能</u>创新，为蓬勃火热的冰雪运动再添场外支撑、踊跃动能，更好地展现了中国高铁新力量。	The Beijing Winter Olympics has set off a trend of ice and snow sports, and China's high-speed rail, with its **<u>smart</u>** upgrade and **<u>intelligent</u>** innovation, adds off-field support and dynamic momentum to the booming ice and snow sports, better showcasing the new strength of China's high-speed rail.	China Daily, 2022/2/9
	科技赋能生活、<u>智能</u>融入未来的理念有望更近人心，科技冬奥也将加速智能机器人的设计理念和技术细节的迭代更新。	The concept of technology empowering life and **<u>intelligent</u>** integration into the future is expected to win more hearts, and the technological Winter Olympics will also accelerate the iterative updates of the design concept and technical details of smart robots.	China Daily, 2022/2/14
Cultural Splendor	首钢大跳台的曲线设计灵感来源于敦煌壁画中的“飞天”造型。飞天飘带造型灵动的曲线与大跳台运动员的运动轨迹相似，并且“飞天”一词与“Big Air”都有向空中腾跃的意思，首钢滑雪大跳台也因此被称为“<u>雪飞天</u>”。	The curvilinear design of the Shougang Big Air is inspired by the "Feitian" (Flying Apsaras) motif in Dunhuang murals. The dynamic curves of the Feitian's ribbon are similar to the trajectory of the Big Air athletes, and the term "Feitian," like "Big Air," conveys the idea of leaping into the air, which is why the Shougang Ski Jumping Platform is also called "**<u>Snow Feitian</u>**."	China Daily, 2022/2/8
	中国人历来讲求“一诺千金”。“言必信，行必果”，就出自于《论语·子路》。这种风范植根于源远流长的中华文化，成为中国同世界交往的价值<u>底色</u>。	Chinese people have always valued a promise worth a thousand pieces of gold. The saying "One's word must be trustworthy, and one's actions must be resolute" comes from the "Analects of Confucius - Zilu." This style is rooted in the long-standing Chinese culture and has become the **<u>underlying color</u>** of value during China's interactions with the world.	People’s Daily, 2022/2/4
	锚定“环境正影响”“区域新发展”“生活更美好”三大目标，北京冬奥会申办、筹办、举办全过程践行《奥	Aiming at the three major goals of "positive environmental impact," "new regional development," and "better life," the Beijing Winter Olympics, in its entire process of bidding, preparing, and hosting, has	People’s Daily, 2022/2/7

	林匹克 2020 议程》，执科技之笔勾勒出一幅壮美的冰雪新**画卷**。	practiced the "Olympic Agenda 2020," using the pen of technology to outline a magnificent new **picture** of ice and snow.	
	国家雪车雪橇中心“**雪游龙**”蜿蜒灵动，将现代奥林匹克元素与中国传统文化完美结合，已成为地标性建筑。	The National Bobsled and Luge Center, known as "**Snow Dragon**," is winding and vibrant, perfectly combining modern Olympic elements with traditional Chinese culture, and has become a landmark building.	Xinhua Net, 2022/2/10
	一场突如其来的瑞雪让国家高山滑雪中心“**雪飞燕**”充满魔力，瑞士名将、平昌冬奥会高山滑雪女子全能冠军米歇尔·吉辛成功卫冕。	A sudden auspicious snowfall has imbued the National Alpine Skiing Center, known as "**Snow Swallow**," with magic, where Swiss veteran and PyeongChang Winter Olympics Alpine Skiing Women's Combined champion Michelle Gisin successfully defended her title.	Xinhua Net，2022/2/17

Table S4 Examples of metaphorical sentences and English translations of the concept of sustainable development

Sub-topic	Metaphorical Sentences	English Translations	Source
Resource Recycling	北京赛区 13 个场馆有 11 个是当年夏季奥运会的'**遗产**'"……比赛场馆反复利用、综合利用、持久利用，这些"中国经验"将是留给中国和世界的一份宝贵财富。	Out of 13 venues in the Beijing area, 11 are considered '**legacy**' venues from the previous Summer Olympics... The repeated, integrated, and sustainable use of these competition venues represents valuable 'Chinese experience' that will be a precious asset left for China and the world.	People's Daily, 2022/2/4
	冰天雪地也是**金山银山**，乘上冬奥快车，崇礼的冰雪产业发展迎来重要机遇，从大山里的小镇，成为全球聚焦的滑雪胜地。	The icy and snowy landscape is also **a mountain of gold and silver**; boarding the express train of the Winter Olympics, Chongli's ice and snow industry has ushered in significant opportunities, transforming from a small mountain town to a globally focused skiing destination.	People's Daily, 2022/2/14
	2008 年奥运会场馆经过改造再利用**变身**"双奥场馆"，这种场馆利用模式，汇集了往届奥运会在可持续方面的优点，为未来的奥运会提供了借鉴。	The venues from the 2008 Olympic Games, after renovation and reuse, have **shapeshifted** into 'dual Olympics venues'; this model of venue utilization has pooled the sustainable advantages of previous Olympic Games, providing a reference for future Olympic Games.	People's Daily, 2022/2/10
	国家游泳中心完成了从"水立方"到"冰立方"的华丽**转身**。该场馆将在闭幕式后成为一个多用途的体育场馆。	The National Aquatics Center has completed a **graceful turn** from the 'Water Cube' to the 'Ice Cube'. This venue will become a multi-purpose sports facility after the closing ceremony.	China Daily, 2022/2/18
	北京冬奥会的竞赛场馆全部采用了绿色技术，100%使用绿色能源，体现出中国以冬奥为契机，积极引导人民群众开启绿色**低碳**生活模式，树立绿色**低碳**文	All the competition venues of the Beijing Winter Olympics have adopted green technologies and use 100% green energy, reflecting China's initiative to use the Winter Olympics as an opportunity to actively guide the public to start a green, **low-carbon** lifestyle,	China Daily，2022/2/17

Environmental Protection	明观，助力中国碳达峰碳中和宏伟目标的实现。	establish a green, **low-carbon** civilization concept, and support the achievement of China's ambitious goals of carbon peak and carbon neutrality.	
	零碳排、**低能耗**、全热回收、无污染，冬奥会制冰造雪的新技术，代表着绿色、科技、环保的奥运理念，是北京冬奥会最美的底色。	Zero carbon emissions, **low energy consumption**, full heat recovery, and pollution-free, the new technology for ice and snow making at the Winter Olympics represents the green, technological, and environmentally friendly Olympic concept, and is the most beautiful background color of the Beijing Winter Olympics.	China Daily, 2022/2/18
	北京冬奥会的绿色理念与实践，不仅为冬奥会举办树立起新的标杆，更是为可持续发展和生态环境保护留下绿色遗产，绿色低碳理念会在这片土地上**生根发芽**。	The green philosophy and practices of the Beijing Winter Olympics have not only set a new benchmark for hosting the Winter Olympics but also left a green legacy for sustainable development and ecological and environmental protection; the concept of green, low-carbon will **take root and sprout** on this land.	China Daily, 2022/2/17
	“绿色、共享、开放、廉洁”的办奥理念落地**生根**，两地三赛区交出冬奥会筹办和本地发展两份优异答卷。	The Olympic concept of "green, shared, open, and clean" has **taken root**, with three competition zones within two regions delivering two excellent reports to both the preparation of the Winter Olympics and local development.	Xinhua Net, 2022/2/4
